# Algae communication, conspecific and interspecific: the concepts of phycosphere and algal-bacteria consortia in a photobioreactor (PBR)

**DOI:** 10.1080/15592324.2022.2148371

**Published:** 2023-03-19

**Authors:** Sergio Mugnai, Natalia Derossi, Yogi Hendlin

**Affiliations:** a Erasmus University College, Rotterdam, Netherlands; b École des Hautes Études en Sciences Sociales, Paris, France; c Erasmus School of Philosophy, Erasmus University, Rotterdam, Netherlands

**Keywords:** bioreactors, consortia, interspecies communication, microalgae, phycosphere

## Abstract

Microalgae in the wild often form consortia with other species promoting their own health and resource foraging opportunities. The recent application of microalgae cultivation and deployment in commercial photobioreactors (PBR) so far has focussed on single species of algae, resulting in multi-species consortia being largely unexplored. Reviewing the current status of PBR ecological habitat, this article argues in favor of further investigation into algal communication with conspecifics and interspecifics, including other strains of microalgae and bacteria. These mutualistic species form the ‘phycosphere’: the microenvironment surrounding microalgal cells, potentiating the production of certain metabolites through biochemical interaction with cohabitating microorganisms. A better understanding of the phycosphere could lead to novel PBR configurations, capable of incorporating algal-microbial consortia, potentially proving more effective than single-species algal systems.

## 1. Introduction

Microalgae are critical parts of aquatic ecosystems that power food webs and biogeochemical cycling. These organisms are considered highly adapted to these environments. This is due to their flexibility in maintaining homeostasis despite a constant influx of foreign material and changing solution composition. Recent research has brought increasing attention to how microalgae interact with their external environment. In fact, wild water environments are extremely variable in terms of physicochemical parameters as well as biological populations, given the many (micro)organisms species populating it.^1^

In terms of their industrial applications, microalgae are currently considered an irreplaceable asset to produce chemicals such as biofuels and pharmaceuticals, or to clean polluted wastewater and sewage through their filtering ability. The most implemented setup to reach these targets currently involves allowing the growth of a specific, single microalgal strain in a protected environment called photobioreactor (PBR). The PBR as a closed system allows constant monitoring of some physical and chemical parameters essential for commercial production, such as light, pH, nitrogen concentration and stirring rates.^2,3^ However, recent studies highlight the importance (and complication) of mimicking a natural ecosystem in such a setup, suggesting the need to arrange the presence of multiple species together in biological consortia.^4,5^ Consortia can potentiate enhanced algal metabolism resulting from interaction between different species, as well as altering growth patterns and productivity of microalgae. For example, many bacteria influence the development of algal blooms in nature,^6^ facilitating the removal of biodegradable organic matter. For this reason, microalgae–bacteria consortia are often deployed in wastewater treatment ponds.^7^ Since interactions between microalgae and bacteria exist in natural habitats, disrupting these multi-species signaling processes may undermine certain desired capabilities or characteristics of the microalgae. Such disruptions may in turn complicate healthy microalgae growth. This is especially the case when growth is attempted in confined environments such as bioreactors.^8^ Investigating the role of interspecies communication in different microalgal species, and between microalgae and bacteria species, is thus crucial to both understand possible photobioreactors enhancements, as well as delineate the limits of closed systems. This paper addresses the role of microalgae communication in the phycosphere, suggesting that such organisms exist necessarily in rich multispecies consortia, which when removed, introduce novel problems for maintaining healthy microalgal growth and require inordinate additional inputs.

## 2. Communication and interaction between microalgae and bacteria

Intraspecific communication between microalgae has been mostly investigated in relation to sexuality and mating.^9^ However, relatively little is known regarding conspecific microalgae communications in response to stress cues within their community. Nor do we yet have adequate knowledge about microalgal capacities for establishing communities and colonies via effective chemical and electrical communication to survive hostile situations or dysbiosis. Indeed, the cultivation of algae in confined environments such as photobioreactors poses challenges to the ecology of microalgae, in terms of how individuals connect with other organisms. So far, most of the research conducted in this field documents issues arising from artificial microalgal extractive systems. But if we aim to explore the behavior of microalgae in their native environment, understanding their web of interactions becomes more challenging since little research directly examines the communicative elements of microalgae in nature and the minimal biotic interactions necessary (and how these regulate microalgal colonies) for interspecifics to thrive.^10,11^ Particularly, the role of colonial health and communication supervene upon individual microalgal cell growth and consequent environmental adaptation of growth rate and metabolism remains largely unexplored.

It is widely established that chemical communication among organisms, either intraspecific and interspecific, requires complex biological structures, including genes, RNA, proteins and other chemical messengers.^12^ Significantly, the *Chara* genus of algae are the first non-animal species where production of electrical action potentials in response to environmental modifications have been detected.^13,14^ From an evolutionary standpoint, (micro)algae possess ancient molecular pathways, such as the presence of heterometric G-proteins and the regulatory protein RGS,^15^ specific four-domain voltage-dependent Ca^2+^ channels^16^ and the TOR signaling network.^17^ These pathways are widely used by land plants, showing that algae can in some applications be employed as models for investigating complex mechanisms found in the plant kingdom, thanks to their simpler anatomy, structure, and metabolic processes. Biochemical communication appears to be a primitive ability of these organisms, essential for establishing communities and colonies.

While intra- and interspecific communication appears to primarily involve allelochemicals, the perception and production of sound in microalgae is an equally significant phenomenon. Algal cells respond variously to indicative sounds in addition to mechanical stresses such as shear stress, hydrostatic pressure, or modifications in the plasma-membrane tension. A small but increasing body of evidence suggests the promising use of algal bioacoustics to promote the growth, cohesion, and productivity of algal organisms.^18,^^19^ Algal bioacoustics measure the intensities and frequencies which may enhance algal growth differentially across species. More experimental work is needed to investigate the most effective combinations, and how algal bioacoustics instigated, are produced, and received in different consortia compositions and ratios.^20^

Microalgae are particularly effective in creating marine communities. They constantly interact with their surrounding environment, in response to physical stimuli such as temperature, chemical gradients, gravity, light and flow shear, which influence the motion of individual swimming microorganisms. Since these microorganisms rarely live individually, environmental influences are reflected at the level of a population, causing cells to aggregate and interact to respond in a more effective way to environmental disturbances. They form what we call ‘algae colonies’, which have the common attribute of being two or more species of algae associating regularly such that this association is recognized as the morphological type of a genus and species.^21^ These patterned groupings can be therefore considered as a form of social behavior.^22^ For instance, recent research highlights the capacity of microalgae to exhibit collective behavior such as swarming,^23,24^ where complex networks are established among individual cells, leading to a coordinated movement flow similar to the swarming of insects or birds’ murmuration. However, the most studied and known phenomenon regarding collective behavior in the algal world is the development of the so-called ‘algal bloom’, some of them being harmful for human populations and ecosystems in general.^25^

As mentioned, microalgae rarely live alone, and many species of microalgae often co-colonize a specific ecosystem. Investigating how different species of microalgae communicate among each other is the basis for analyzing the relationships within an aquatic ecosystem. Such knowledge is necessary to improve the growth and health of microalgal communities inside confined environments such as PBRs. Due to the competition for resources, the most obvious example of communication between different species of microalgae is allelopathy. Allelopathy (i.e. inhibition) is commonly defined as the process involving chemical compounds released into the surrounding medium that have adverse effects, either directly or indirectly, on the growth of microorganisms.^26^ The outcome of this type of communication can include death, paralysis (for motile cells) or inhibition of the receivers’ growth.^27^ Through these mechanisms, algal cells can exert various forms of control over competitive species. Many mechanisms exist. For instance, allelochemicals can impair photosynthesis by affecting protein complexes such as phycobilisomes^28^ or blocking the electron transfer in algal photosystems.^29^ The cell membrane is both a barrier and medium of matter and energy exchange between the cell and its external environment. Damage to the cellular membrane by allelochemicals provokes severe consequences for a cell’s fluidity and selectivity, negatively affecting its vitality and functioning.^30^ Other physiological aspects on which allelopathy can play a role include the enzyme activity of cells,^31^ and DNA translation and transcription.^32^

Mutualistic interactions between microalgae and other microorganisms such as bacteria, conversely, yield equally interesting results. Scientific interest in cooperation between microalgae and bacteria has grown considerably in the last decade, especially due to the practical applications of understanding microalgal-bacterial consortia beneficial for industrial activities such as wastewater treatment or biofuel production.^33,34^ For instance, Amin et al.’s ecological studies^35^ identify specific groups of heterotrophic bacteria capable of establishing close associations with specific microalgae, influencing microalgal behaviors in various ways, from the stimulation of growth and morphogenesis to the germination of spores and colonization by forming biofilm communities.^36^ In addition to biofilm bacteria, microalgae can secrete exudates to influence heterotrophic bacteria (sometimes also called planktonic organisms) in their surroundings. For this reason, the term ‘*phycosphere’* has been coined to describe the region of influence of microalgal exudates upon co-occurring organisms.^37,38^ The phycosphere is discussed further below. Another form of interaction between bacteria and microalgae is signal transduction. Here, the involved chemicals are not mere nutrients, but also precipitate specific abilities such as activation/inhibition of gene expression and/or physiological activities. Signal transduction between algae and bacteria goes beyond the typical boundaries set by systematic biology, often classified as ‘interkingdom signaling’. In microalgae/bacteria mutualism, bacteria can secrete chemical signals that induce growth and morphogenesis of algae,^39^ while microalgae can repress the formation of excess biofilms (biofouling) on bacterial surfaces.

Algal chemical secretions can also result in downregulating bacterial quorum sensing [QS,^40^]. QS in this case involves the coordination of bacterial gene expression in a population density-dependent manner via the production and exchange of specific signal substances between individual interspecific cells. Algae are capable of interfering with this communication mechanism, thus repressing the growth and development of bacterial communities by mimicking bacterial QS signals, such as AHL [acyl-homoserine lactone,^41,42^].

The last type of microalgae and bacteria mutualism is horizontal gene transfer (HGT). Horizontal gene transfer is an evolutionary process, in which genes are horizontally transferred between adjacent organisms, for example, microalgae and bacteria living in the phycosphere.^43,44^ HGT generally refers to any DNA (“vertical”) transmission that is not from parent to offspring (reproduction). HGT occurs by three well-understood genetic mechanisms:^45^ *transformation* (bacteria take up DNA from their environment), *conjugation* (bacteria directly transfer genes to another cell, such as a microalgal cell) and *transduction* (bacteriophages move genes from one cell to another). The ecological advantage of such a feature is that horizontally transferred genes confer microalgae key functions to better survive under stress. For instance, microalgal-associated microbial communities are often enriched in gene functions involved in vitamin synthesis, the detection and attachment to host surfaces, biofilm formation, polysaccharide catabolism and various defense mechanisms.^46^

On the other hand, microalgae can also be detrimental to other organisms. Microalgae can release compounds harmful to their grazers^47,48^ or to non‐algal competitors for the same resources, such as bacteria and fungi.^49–51^ The possibility cannot be excluded that microalgae are involved in chemical interactions with multiple actors (i.e. ‘multitrophic’ interactions), for instance by producing compounds beneficial for individuals whose presence is disadvantageous for grazers, as observed for other organisms.^52–55^

To conclude, individual microalgae communicate with other conspecifics, with other (micro)algal species, and with other organisms in beneficial or detrimental ways which construct their individual, species, and group niches, increasing fitness and creating conducive ecologies.

## 3. The role of the phycosphere

As mentioned in the previous section, most of the interactions between microalgae and bacteria only affect the algal cell’s external layer. To compare with terrestrial soil systems, it is well known how the contact zone surrounding a root is crucial for determining the health and the metabolism of the entire plant, and for the continued functioning of the soil ecosystem. This narrow zone surrounding – and influenced by – plant roots is named the *rhizosphere* and is considered as one of the most complex ecosystems on Earth, hosting microbial communities such as fungi and bacteria that play fundamental roles in the plant growth and metabolism.^56^ From this perspective, soil is not seen as a mere container for the root system, simply providing support, water and nutrients, but a complex ecosystem in which an intricate network of living organisms enables emerging interspecies relationships.

Despite the highly divergent media characteristics between soil and water, the concept of the rhizosphere does translate from plant ecology to any aquatic environment hosting microalgae. Recently, a new term – *phycosphere* – has been coined to define the microenvironment immediately surrounding microalgal cells. This area determines which metabolites are readily available and the parameters for interactions between microalgae and other microorganisms (mainly bacteria).^57^ This interaction allows chemical exchanges between bacteria and microalgae. The latter continuously exudate organic matter (up to 50% of the photosynthates), beneficial bacteria produce metabolites essential to algal growth and metabolism. Understanding this interface is of the utmost importance to figure how global biogeochemical fluxes and oceanic nutrient fluxes work.^57,58^ As already anticipated when discussing communication, the relationship between microalgae and bacteria is mediated and regulated by quorum sensing (QR), a process by which bacteria coordinate their gene expression and metabolism at the population level, and by quorum quenching (QQ), which suppresses the activity of antagonistic bacteria.^59,^ In the phycosphere a delicate equilibrium between promoting and suppressing actions is established, regulating the ecosystem community in terms of mutualistic and parasitic behavior.

The moment we consider photobioreactors (PBRs) as an aquatic ecosystem, even though at a small scale, the interactions in the phycosphere must be taken into consideration, especially when a polycultural approach is adopted. Associated with microalgal cultivation and PBRs, polycultures convey a community of diverse algal and bacterial species living in ecological homeostasis. Why adopt the term polyculture for microalgae? To date, microalgal cultivation has mainly focused on exploiting monocultures of highly productive microalgal strains.^60–62^ However, microalgal monocultures are difficult to maintain due to several constraints such as accidental contamination by wild microalgal strains, bacteria and pathogens.^63–65^ Recent research suggests that polycultures can promote both ecosystem robustness and productivity [for example, see ^33^]. For this reason, a greater understanding of species interactions along with patterns of communities’ change with time in a confined environment is needed to enhance the ecological health and the productivity of PBR polycultures.

To fully understand the issue at stake, an obvious comparison can be made between PBRs and another important ecosystem, the agrarian one. Briefly overviewing monoculture versus polyculture systems in agriculture can provide possible implications of such different systems in algal cultivation. For terrestrial agriculture, monoculture systems involve growing a single (usually staple) crop in a field during the growing season. Monoculture production systems simplify crop management and allow for concentration of efforts and resources on maximizing economic return from a single crop. On the other hand, these systems do not reproduce complex natural ecosystems because they tend to reduce soil biodiversity as well as pauperize the soil, due to increased soil erosion, greater nutrient leaching and lower water-holding capacity.

On the contrary, polyculture is the growing of two or more crops together on the same piece of land during a growing season. Polyculture allows for spatial diversification of plant species, providing greater opportunity to efficiently use soil and environmental resources compared to monoculture. Polyculture systems in some cases can be challenging to manage because species growing together compete and have diverse resource needs. But mostly, they are more time intensive to harvest, resisting easily mechanized collection. Polycultures, however, have the potential to provide several advantages compared to monocultures, the most important ones being a greater tolerance to environmental and pest stress, providing insurance against total crop failure, and requiring and providing differential nutrients and rooting characteristics which have the potential for greater exploitation of available light, water, and nutrient resources. In soil, polycultures take advantage of the complex relationships between different plant species and microorganisms, with distinct and diversified arbuscular mycorrhizal (AM) fungal community composition,^66^ symbionts aiding in plant nutrient acquisition, drought tolerance, pathogen protection, water uptake, and numerous other functions that affect plant health.^67^ Overall, polycultures open up the possibility to consider algal interactions beyond a framework of resource scarcity within which organisms are bound to compete.

The question therefore is can a polyculture approach be applied to PBRs, and how could it benefit microalgal cultivation? Aquatic polyculture cultivation increases productivity of microalgae via both resource use efficiency and community stability.^68,33^ Multiple microalgal species occupying different functional niches make use of the resources in a more efficient and complementary way due to their different light absorption spectra, nutrient requirements, uptake rates and physiological complementarity.^69^ When algal communities contain multiple species, they become more stable under varying conditions compared to monocultures’ disturbance-reduced productivity. Stability allows for increased autonomy and resilience against molecule accumulation and microbial contamination, which represent considerable issues especially when production needs to be scaled up,^70^ and production rates increased.^71^ These co-benefits reduce maintenance costs as well as downtime. Concomitantly, however, extensive knowledge of the whole community’s functioning, planning and onset of optimal community co-habitant composition, are required. Additionally, these benefits come with the proviso that polycultures may only be suitable for a set of well-studied conditions. From a different vantage point, the polyculture concept can be interpreted as associating microalgae and bacteria instead of different strains or species of microalgae alone. Through this lens, the concept of phycosphere previously introduced suggests a positive interaction between these two groups of organisms, an interaction possibly capable of enhancing the productivity of PBRs by mimicking – however reductively – a wild aquatic ecosystem.

The role the phycosphere in PRBs remains at the initial stages of long-term scientific investigation, but already promising results are indicated (Table 1). For instance, Feng et al. (2021) found that the removal of pollutants from anaerobic digestion effluents is dramatically enhanced when algal-bacteria consortia rather than monocultures are introduced.^72^ Chaiwong et al. (2018) investigated the treatment performance of an algal-bacterial PBR (AB-PBR) treating a septic tank effluent compared to a solely algal one.^73^ Here, the efficiency and effectiveness of the AB-PBR was higher than a traditional single algal strain one. Several studies have proven the possibility to improve H_2_ production when using co-cultures of algae and bacteria, with some of them focusing on the use of the alga *Chlamydomonas* [reviewed by ^75^]. Bélanger-Lépine et al. (2020) found that a native microalgae-bacteria consortium isolated locally grew better in wastewater when compared to pure algal strains, and that different fatty acid profiles were produced.^76^ Similar results were obtained for Fito and Alemu’s (2018) evaluation of the potential of an algal-bacteria consortium to manage municipal wastewater.^77^ Wastewater treatment and removal of pollutants have been the major fields of research until now regarding the potential for algae-bacteria consortia. In the past years, investigation of microalgal-bacterial consortia for biotechnological applications has been promoted. Even though the physiological mechanisms behind the interaction between microalgae and bacteria in practical applications are not yet well understood, promising results suggest increased capacities for water cleaning. Zhang et al. (2020) attentively reviewed these aspects, including finding improved capacity for carbon and nutrient removal by enhanced flocculation, heavy metal removal via biosorption and adsorption, degradation of organic hazardous compounds, and even production of biofuels.^78–82^

**Table 1.** Recent developments of algae-bacteria consortia in a photobioreactor (PBR).

Purpose	Algal species	Bacterial species	Reference
Biofuel production	*Chlorella* sp., *Scenedesmus* sp., *Ulothrix* sp., *Klebsormidium* sp.	Rhodocyclaceae, Xanthomodaceae, Rhodobacteraceae,	Reviewed by ^77^
]Biogas slurry treatment	*Chlorella vulgaris* strain FACHB-8	*Shinella* sp. strain YHB03	^74^
H_2_ production	*Chlamydomonas reinhardtii*	*Bradyrhizobium japonicum, Pseudomonas* sp. strain D, *Escherichia coli, Bacillus subtilis, Herbaspirillum* sp., *Rhodosprillum rubrum, Stenotrophomonas* sp., *Phyllobacterium* sp., *Thuomonas intermedia*	Reviewed by ^76^
Wastewater treatment	*Chlorella* sp. and Trebouxiophyceae	Proteobacteria and Verrucomicrobia	^61^
	*Chlorella* sp., *Synechocystis* sp., *Phormidium* sp. and *Monoraphidium* sp.	Proteobacteria, Bacteroidetes, Deinococcus-Thermus and Firmicutes	^4^
	*Chlorella* *zofingiensis*	Proteobacteria, Bacteroidetes, Actinobacteria, Acidobacteria, Nitrospira and Chloroflexi	^74^
	*Chlamydomonas* sp., *Chlorella* sp. and *Scenedesmus* sp.	Naturally existing municipal wastewater bacteria	^4^
	*Chlorella* sp., *Euglena gracilis, Chlorella pyrenoidosa, Scenedesmus* sp. 336	*Acinetobacter* sp., *Rhizobium* sp., *Bacillus firmus, Beijerinckia fluminensis, Emticicia* sp. EG3, *Kluyvera* sp., *Bacillus licheniformis, Exiguobacterium* sp.	^79^
	*Chlorella* sp. and *Scenedesmus* sp.	Proteobacteria, Cyanobacteria, Bacteroidetes, Chloroflexi and Nitrospirae	^36^

## 4. Conclusions

Photoautotroph–bacteria co-culture investigations so far indicate higher success rates in societal applications and algal health than monocultures. Algal management in PBRs thus far in commercial units have mostly sought laboratory conditions with single species. This has left experiments with multiple algal strand polycultures, let alone algal-bacterial consortia, largely unexplored. Yet, even the extant research on these axenic interspecies consortia for commercial applications likely underestimates the true extent of necessary symbionts which allow algae to perform their water cleaning ecosystem services during metabolism. Other organisms besides bacteria may also be indispensable for optimal algal health and productivity – part of the phycosphere that deserves additional analysis. Reproducing *in situ* environmental conditions in closed laboratory systems is always difficult with organisms. Such enterprises can be additionally challenging with species like algae that are constitutively multi-species. Nonetheless, recent experiments show the effective mobilization of interspecies algal-bacteria PBR to better tackle real-world applications. Of course, the particular goal for *ex situ* microalgal consortia cultivation (e.g., producing eicosapentaenoic acid versus water remediation) influences the necessity to attend to a fuller or narrower classification of co-symbionts. Through better understanding the phycosphere, more sophisticated forms of PBRs incorporating algal-microbial consortia may require fewer inputs and be more productive.
